# Type and effectiveness of community-based interventions in improving knowledge related to cardiovascular diseases and risk factors: A systematic review

**DOI:** 10.1016/j.ajpc.2022.100341

**Published:** 2022-04-06

**Authors:** Hamid Yimam Hassen, Rawlance Ndejjo, Jean-Pierre Van Geertruyden, Geofrey Musinguzi, Steven Abrams, Hilde Bastiaens

**Affiliations:** a Department of Population Health and Family Medicine, Faculty of Medicine and Health Sciences, University of Antwerp, Doornstraat 331 Wilrijk, Antwerp 2610, Belgium; b Global Health Institute, Faculty of Medicine and Health Sciences, University of Antwerp, Antwerp 2610, Belgium; c Interuniversity Institute for Biostatistics and Statistical Bioinformatics, Data Science Institute, Hasselt University, Diepenbeek 3590, Belgium; d Department of Disease Control and Environmental Health, School of Public Health, Makerere University, Kampala, Uganda

## Abstract

Background: Despite an improvement in the healthcare system, cardiovascular diseases (CVDs) remain the leading cause of morbidity and mortality worldwide. Improving knowledge is a key for behavioral change towards prevention of CVDs. However, up-to-date evidence is limited on the effect of interventions on CVD knowledge. Thus this study aimed to synthesize comprehensive evidence on the type and effectiveness of community-based interventions (CBIs) to improve knowledge related to CVDs. Methods: We performed a systematic review of studies that tested the effectiveness of CBIs in improving CVD knowledge. International databases including MEDLINE, EMBASE, CINAHL, PSYCINFO and Cochrane register of controlled studies were searched for studies published between January 2000 and December 2019. The Cochrane risk of bias tools were used to assess the methodological quality of included studies. Since CVD knowledge was measured using various tools, results were synthesized narratively and reported in line with the reporting guideline for Synthesis Without Meta-analysis (SWiM). The review protocol is registered in the PROSPERO database (CRD42019119885). Results: 7 randomized and 9 non-randomized controlled trials involving 34,845 participants were included. Most of the interventions targeted the general population and majorities delivered the intervention to groups of individuals. Likewise, most of the interventions employed various intervention components including health education using different strategies. Overall, most studies showed that CBIs significantly improved knowledge related to CVDs. Conclusion: Community-based CVD preventive interventions are effective in improving knowledge related to CVD and risk factors. Measures to scale up CBIs are recommended to improve an individual's level of CVD knowledge, which potentially helps to counter the growing burden of CVDs.

## Graphical abstract

**Figure:**
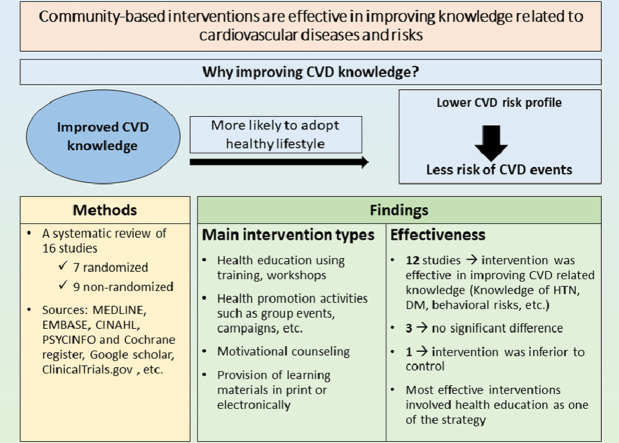

## 1 Introduction

The World Health Organization (WHO) predicts, non-communicable diseases (NCDs) are expected to cause above three-fourth of all global deaths in 2030 [1]. In particular, cardiovascular diseases (CVDs) are the leading cause of disease burden accounting for an estimated 523 million cases and 18.6 million deaths in 2019 [2]. In the past three decades, most high-income (HIC) and some middle-income countries showed a steady decline in the age-standardized mortality rate due to CVDs [3]. In contrast, the burden increased in most low- and middle-income countries (LMICs) contributing today to 75% of all global CVD deaths [4]. Recently, the age-standardized rate of CVD death has begun to increase in some areas where it was previously declining [2]. Acquisition of lifestyle-related risk factors due to demographic changes, socioeconomic and epidemiological transitions, and the influence of globalization and industrialization could be the causes of such huge variations in the CVD burden and trends across time and contexts [3], [4], [5].

An individual's lifestyle including dietary habits, tobacco use, level of physical activity (PA), excessive alcohol consumption, and stress greatly determines the occurrence of CVDs [6,7]. Likewise, metabolic and physical risk factors including high low-density lipoprotein (LDL) cholesterol, high body mass index (BMI), hypertension and diabetes contribute to a large proportion of CVD morbidity and mortality globally [2]. Knowledge of behavioral and metabolic risks is the central element to adopt healthy lifestyles [8], [9], [10]. However, knowledge and awareness related to CVDs and their risk factors is still low [11]. Therefore, improving an individual's knowledge level related to CVD and risk factors is an essential element of CVD prevention and control programs [12,13]. The burden of CVD and risk factors can be reduced in the entire community using community-based interventions (CBI) aimed at improving CVD knowledge and multi-component risk reduction practices [14], [15], [16]. Health promotion and disease preventive interventions in the community as well as in primary care settings seem effective in improving CVD risk factors and estimated risk scores [17,18]. Lifestyle interventions using various strategies including health education seem more cost effective than pharmacological interventions in resource limited LMICs as well as HICs [19], [20], [21].

Interventions targeting the general population and/or high risk groups have been developed and their impact on CVD knowledge and change in behavior has been tested. A review of studies before 2015 [22] indicated that CBIs enhanced short-term knowledge related to CVD risk factors, though the review was limited to interventions in non-urban settings. A comprehensive up-to-date synthesized evidence is limited on the long term effect of such interventions on CVD related knowledge while such exhaustive information is crucial to inform prevention and control efforts across different contexts. Available reviews give little attention to CVD related knowledge and are limited to specific target populations such as prisoners or vulnerable groups [23,24], region [25,26] or context such as only rural areas [22]. Besides, the variation in effectiveness across different intervention approaches and strategies, target populations and contexts is not well documented. Therefore, we synthesized the type of intervention approach and components as well as their effectiveness in increasing knowledge on CVD, their risk factors and preventive mechanisms. Furthermore, we compared the intervention effectiveness across different contexts. The evidence from this review is beneficial for public health practitioners and the scientific community to scale up effective interventions to enhance prevention and control of CVDs.

## 2 Methods

This review is part of the SPICES project - Scaling-up Packages of Interventions for Cardiovascular diseases in selected sites in Europe and Sub-Saharan Africa (https://www.uantwerpen.be/en/projects/spices/). The review aimed to synthesize available evidence on CBIs targeting CVD risk knowledge and behaviors including smoking, PA level, dietary habit, and alcohol intake. In this study, we particularly focused on studies that reported CVD knowledge as one of the outcomes. The review protocol is registered in the PROSPERO International prospective register of systematic reviews (Reg. no.: CRD42019119885). Since this study is a systematic review of published data, it was exempted from the institutional board review. Details of the search strategy and screening process is available elsewhere [27]. Methods specific to this paper are briefly summarized below.

### 2.1 Search strategy

Electronic databases including MEDLINE, CINAHL, EMBASE, Cochrane register of controlled studies, and PSYCINFO were searched. In addition, we checked databases including thesis online, OpenGrey, ProQuest, CHW Central, Google Scholar, ClinicalTrials.gov and the WHO International Clinical trials registry platform. We developed a comprehensive search strategy based on terms related to the population, intervention and outcomes of interest. The search strategy used in the MEDLINE is available in the Supplementary Material (Box S1). Furthermore, reference lists of included articles were searched and eligible studies were incorporated in this review.

### 2.2 Study selection

Studies were included if they tested interventions to prevent CVDs and reported knowledge related to CVD and/or risk factors as an outcome. Studies being the subject of multiple publications were considered as a single study. Studies were included if they: were published between the years inclusive of 2000 and 2019; were either primordial or primary prevention of CVD; and interventions were based in the community including workplaces, households, schools, sport centers, religious centers, pharmacies, primary health care units, etc. Studies were excluded from this review if; study participants were of individuals who had a formal diagnosis of any type of CVD; interventions involved clinical procedures and/or pharmacologic components and/or solely took place in clinical settings; sample size was less than 150, attrition rate more than 40%, the follow-up duration less than 9 months; or if analyses included individuals aged below 18 years. Individual or cluster-randomized controlled or controlled quasi-experimental or interrupted time series studies were included. This review was restricted to studies reported in the English language with no limitation on the study location.

Articles from electronic databases were exported as EndNote files where duplicate articles were checked and deleted. Then, to facilitate screening and collaboration, the remaining abstracts were imported into rayyan.QCRI.org [28]. Using structured inclusion and exclusion criteria, double screening (HYH and RN) was performed independently for all retrieved titles/abstracts. Articles selected for full-text review were screened again for final decision of inclusion. When the primary reviewers did not agree concerning the eligibility of an article in the final review, other reviewers (HB and GM) were consulted. The review process including the reasons for exclusion is summarized in the PRISMA flow chart.

### 2.3 Risk of bias assessment

We used the revised Cochrane tool for Risk of Bias (RoB2) in order to assess the risk of bias of individual randomized studies with some additional components for cluster randomized studies [29]. Non-randomized controlled (NRC) studies were evaluated using the Risk of Bias In Non-randomized Studies - of Interventions (ROBINS-I) tool [30]. Two authors (HYH and RN) independently assessed the risk of bias, and any differences were resolved through discussion with a third reviewer (HB).

### 2.4 Data extraction

Double data extraction was done from all eligible full-text articles by two reviewers (HYH and RN). Data related to study design, intervention characteristics and contexts, comparator group(s), detailed participant characteristics, sample size and attrition rate, follow up (FU) duration, outcome measures, result summaries, and funding sources were extracted. Intervention description including components, setting, approach, duration, and intensity were also collected. Furthermore, the outcome measurement tool, effect estimates, and observed changes in the level of CVD knowledge were recorded for each group. Authors were contacted twice via email whenever key information was missing.

### 2.5 Data synthesis

Due to the variation in the outcome measures and intervention types, a formal meta-analysis of effect estimates was not possible for this review and thus, we used the Synthesis Without Meta-analysis (SWiM) reporting guideline to present the results [31] and the checklist is available in the Supplementary Material (Table S2). Data were synthesized narratively and descriptive statistics were considered to summarize the main study characteristics including the risk of bias. Studies were grouped according to the following three criteria: (1) study design (RCT or NRC); (2) target population (general population vs. high-risk); and (3) intervention approach (individual, group or combined). Findings are descriptively presented and discussed by income per capita, intervention approach, study design, and risk of bias. For comparison, data are presented using tables mentioning country and year of study, intervention approach and duration, target population, context, and outcome measures. The findings reported in different eligible studies were expressed either in terms of absolute differences (i.e., in means/medians/proportions) and/or in terms of relative measures (i.e., ratio of prevalence/risks/) between intervention and control groups. Lastly, to find out whether any evidence of an intervention effect on the outcome exists, we synthesized all available evidence in tabular format by vote counting based on the direction of the estimated effects [32].

## 3 Results

**Fig. 1:**
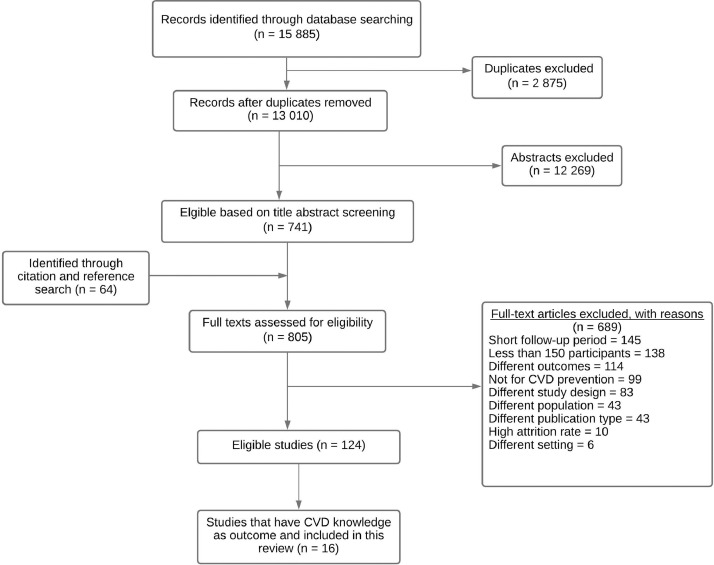
Study selection.

A total of 15,885 abstracts were retrieved from all the databases. We screened the titles and abstracts, and 741 were promoted for full-text review. We identified 64 additional articles through manual searching thereby leading to a total of 805 articles. The full-text review resulted in 124 studies to be eligible. Of those, 16 studies involving 34,845 participants assessed knowledge related to CVD and risk factors as an outcome and were therefore included in this review. The PRISMA flow chart illustrating the screening process is summarized in Fig. 1.

### 3.1 Characteristics of included studies

Detailed characteristics of the included studies are presented in the supplementary material (Table S1). Of the 16 included studies, 9 were conducted in HICs, particularly, three in the USA [33], [34], [35], two in Japan [36,37], one each in Australia [38], Canada [39], UK [40], and the Netherlands [41]. Seven studies were performed in LMICs including four in China [42], [43], [44], [45], and one each in Pakistan [46], India [47] and Bangladesh [48].

Seven studies were randomized trials, of which three were individual-randomized [33,42,45] while four were cluster-randomized studies [34,40,47,48]. Nine studies were NRC before-after studies [[35], [36], [37], [38], [39],^41^,^43^,^44^,^46^]. Of the seven randomized studies, two had low risk of bias [33,40] while five had some concerns of bias [34,42,45,47,48] related to either in the randomization process, selection of participants, deviation from the intervention or missing outcome measurements. In particular, all three individual randomized studies [33,42,45] had bias due to deviation from the intended intervention mainly because of not clearly indicating the possibilities of participants to switch the intervention under study or other similar ongoing interventions. Three out of four cluster-randomized studies had some concerns of bias from the timing of participant recruitment and randomization of clusters. Out of 9 non-randomized studies, two had low risk [38,41], four moderate [35,36,39,43] and three serious risk of bias [37,44,46]. Three out of these 9 studies suffered from serious bias due to lack of comprehensive adjustment for potential confounders. Furthermore, three and four studies had some concerns of bias due to confounding and deviation from intended intervention, respectively. Details of the risk of bias assessment for each of the individual studies is available in the Supplementary Material (Table S3, Fig S1).

Most (*n* = 11) of the interventions targeted the general population [34,[36], [37], [38], [39],^41^,^43^,^44^,[46], [47], [48]] through various primordial and primary prevention activities while few of them targeted specifically high-risk groups including individuals with diabetes [33,40], hypertension [42], or older adults [35,45].

With regard to the approach, majority of studies included in this systematic review delivered the intervention to groups of individuals [33,[35], [36], [37], [38], [39],[41], [42], [43], [44],^46^,^47^], a few of them employed a one-to-one approach [34,40] while some others combined both approaches [40,45,48]. Interventions employed various components including awareness creation and health education in group or one-to-one via lectures, courses, trainings and/or workshops [33,35,36,38,[40], [41], [42], [43], [44], [45], [46],^48^]; health promotion activities through group events, social marketing and campaigns [33,37,38,43,[46], [47], [48]]; individual-based motivational counseling face-to-face or via phone calls [34] or peer support programs; [40] provision of learning materials and/or educational messages in print and/or electronically [39]; and organizational changes [38,41,[43], [44], [45]].

Almost all (*n* = 15) studies had an intervention duration longer than 12 months while one study lasted for only three months. More specifically, four studies implemented the intervention for 12 months [33,34,37,40], six studies for 24 months [36,[41], [42], [43],^47^, ^48^], one study for 18 months [45], and four studies for 36 months [35,38,44,46]. The point of FU assessment ranged from 12 to 36 months, in which some studies assessed the outcome at multiple FU points.

### 3.2 Measures of knowledge related to CVD and risk factors

Studies employed various measures of CVD related knowledge including diabetes knowledge [33,40,48], hypertension knowledge [42], both knowledge of diabetes and high blood pressure [35], knowledge of risk factors and health behaviors related to CVDs [43,44,46,47], knowledge about PA guidelines [36], early signs of stroke [37,39], knowledge of recommended level of fruit & vegetable intake, and portion size [34,38,41], or measurement of a general health knowledge score [45].

### 3.3 Effectiveness of CBIs on knowledge of CVD and risk factors

A summary of the direction of effects of CBIs on various CVD knowledge measures is summarized in Table 1. Overall, in 12 studies, i.e. 4 RCTs and 8 NRC studies, the improvement in knowledge related to CVDs and/or risk factors is significantly higher in the intervention group as compared to controls [33,[35], [36], [37], [38], [39],^41^,^42^,[44], [45], [46],^48^]. Whereas three studies found no significant difference in the average post-intervention knowledge level across intervention groups [34,40,47] and all of them were cluster-randomized studies. One study [43] with some concerns of bias in comparability of clusters and deviation from the intended intervention, showed that the increase in tobacco related knowledge was significantly larger in the control areas (average increase from 5.21 to 6.38) than the intervention (from 4.97 to 5.74). In addition, knowledge related to diet and physical activity also decreased in the intervention group compared to the controls [43].

**Table 1:** Summary of effect of community-based interventions on knowledge related to cardiovascular diseases.

Study ID, country	Study design	Comparison	Target group	Effect on the outcomes	Effect measure	Direction	Effect size	P value
Lu et al. [42], China	RCT (3 arms)	IG: Interactive workshop vs. CG: Self reading learning	High-risk	Hypertension knowledge score	MD	I	3	<0.001
		IG: regular lecture vs. CG: Self reading learning	High-risk	Hypertension knowledge score	MD	I	1.7	<0.001
Chao et al. [45], China	RCT	IG: Community-based health management vs. CG: Usual care	High-risk	Health knowledge score	MD	I	12.14	<0.0001
Brown et al. [33], USA	RCT	IG: Culturally competent diabetes self-management education vs. CG: Wait-listed (control)	High-risk	Diabetes knowledge	MD	I	3.09	<0.0001
Fottrell et al. [48], Bangladesh	C-RCT (3 arms)	IG: Participatory learning and action vs CG: Usual care	General adult population	Ability to report one or more valid cause of diabetes	AOR	I	35.7 (17.7, 71.9)	<0.0001
		IG: mHealth mobile phone messaging vs CG: Usual care	General adult population	Ability to report one or more valid cause of diabetes	AOR	I	3.77 (2.05, 6.91)	<0.0001
		IG: Participatory learning and action vs CG: Usual care	General adult population	Ability to report one or more valid symptom of diabetes	AOR	I	24.0 (11.3, 50.9)	<0.0001
		IG: mHealth mobile phone messaging vs CG: Usual care	General adult population	Ability to report one or more valid symptom of diabetes	AOR	I	4.37 (2.07, 9.24)	<0.0001
		IG: Participatory learning and action vs CG: Usual care	General adult population	Ability to report one or more valid complication of diabetes	AOR	I	35.4 (17.8, 70.4)	<0.0001
		IG: mHealth mobile phone messaging vs CG: Usual care	General adult population	Ability to report one or more valid complication of diabetes	AOR	I	5.42 (2.60, 11.3)	<0.0001
		IG: Participatory learning and action vs CG: Usual care	General adult population	Ability to recognize one or more valid complication of diabetes when prompted	AOR	I	18.3 (7.66, 43.9)	<0.0001
		IG: mHealth mobile phone messaging vs CG: Usual care	General adult population	Ability to recognize one or more valid complication of diabetes when prompted	AOR	I	3.88 (1.47, 10.2)	0.0063
		IG: Participatory learning and action vs CG: Usual care	General adult population	Ability to report one or more valid way to prevent diabetes	AOR	I	10.0 (5.44, 18.5)	<0.0001
		IG: mHealth mobile phone messaging vs CG: Usual care	General adult population	Ability to report one or more valid way to prevent diabetes	AOR	I	4.31 (2.10, 8.85)	0.0001
		IG: Participatory learning and action vs CG: Usual care	General adult population	Ability to report one or more valid way to control diabetes	AOR	I	8.36 (4.42, 15.8)	<0.0001
		IG: mHealth mobile phone messaging vs CG: Usual care	General adult population	Ability to report one or more valid way to control diabetes	AOR	I	3.93 (1.90, 8.12)	0.0002
Joshi et al. [47], India	C-RCT	IG: Health promotion campaign vs. CG: No intervention	General adult population	Knowledge 6 key health behaviors related to CVD	MD	NS	–0.08(−0.14, 0.02)	0.15
Resnicow et al. [34], USA	C-RCT (3 arms)	IG: A self-help intervention with 1 telephone cue call vs. CG: Standard practice	General adult population	Knowledge of portion size	MD	NS	ND	
		IG: A self-help intervention with 1 telephone cue call and 3 counseling calls vs. CG: Standard practice	General adult population	Knowledge of portion size	MD	NS	ND	
Simmons et al. [40], UK	C-RCT (4 arms) - 2 × 2 factorial	IG: Group meeting vs CG: No intervention	High-risk	Diabetes Knowledge	ES	NS	0.17 (−0.17, 0.51)	
		IG: One-to-one vs CG: No intervention	High-risk	Diabetes Knowledge	ES	NS	−0.13 (−0.47, 0.21)	
		IG: Combined vs CG: No intervention	High-risk	Diabetes Knowledge	ES	NS	0.05 (−0.35, 0.45)	
Lv et al. [43], China	NRC	IG: Community Interventions for Health (CIH) vs. Routine practices	General adult population	Tobacco-related knowledge	MD	C	−0.4	S
		IG: Community Interventions for Health (CIH) vs. Routine practices	General adult population	Diet related knowledge	MD	NS	−0.3	NS
		IG: Community Interventions for Health (CIH) vs. Routine practices	General adult population	PA-related knowledge	MD	NS	−0.34	NS
Saito et al. [36], Japan	NRC	IG: Community wide intervention vs. CG: Standard health promotion service	General adult population	Awareness and PA guideline knowledge	APD	I	0.82 (0.33, 1.31)	<0.01
Glasson et al. [38], Australia	NRC	IG: The Eat It To Beat It program+ vs ongoing Good for Life program CG: only ongoing Good for Life program	General adult population	Understanding of fruit servings recommended each day	PD	I	0.05	S
		IG: The Eat It To Beat It program+ vs ongoing Good for Life program CG: only ongoing Good for Life program	General adult population	Understanding of fruit serving size	PD	NS	−0.02	NS
		IG: The Eat It To Beat It program+ vs ongoing Good for Life program CG: only ongoing Good for Life program	General adult population	Understanding of vegetable servings recommended each day	PD	I	0.01	S
		IG: The Eat It To Beat It program+ vs ongoing Good for Life program CG: only ongoing Good for Life program	General adult population	Understanding of vegetable serving size	PD	NS	0.05	NS
Bertera [35], USA	NRC	IG: Storytelling vs. CG: Assessment only	High-risk	Knowledge, attitudes, and practices related to diabetes and high blood pressure	MD	I	0.282	NS
Silver et al. [39], Canada	NRC (4 arms)	IG: print vs CG: No intervention	General adult population	Ability to name >2 warning signs of stroke	MD	NS	0.2	NS
		IG: low-level TV vs CG: No intervention	General adult population	Ability to name >2 warning signs of stroke	MD	I	0.48	0.021
		IG: high-intensity TV vs CG: No intervention	General adult population	Ability to name >2 warning signs of stroke	MD	I	0.62	<0.001
Nishtar et al. [46], Pakistan	NRC	IG: Community health education vs. CG: No intervention	General adult population	Knowledge about CVDs and their prevention	PD	I	0.16	<0.001
Huang et al. [44], China	NRC	IG: Training of health staff and health education vs CG: No intervention	General adult population	Knowledge and perceptions on HTN, dietary and lifestyle behaviors.	PD	I		<0.05
Kloek et al. [41], Netherlands	NRC	IG: Community health interventions vs CG: Usual care	General adult population	Fruit and vegetable knowledge score	MD	I	0.13	0.03
Miyamatsu et al. [37], Japan	NRC	IG: Television campaign vs. CG: No intervention	General adult population	Knowledge about early symptoms of stroke	PD	I	0.12	<0.05

*Keys: Positive effect (green), evidence of favorable impacts of the intervention; No significant effect (orange), evidence of null impacts of the intervention; Negative (red), the control group is better than the intervention.*

*AOR: Adjusted Odds Ratio; CG: Control Group; ES: Effect size; IG: Intervention Group; MD: mean difference; ND: No Data; PD: Proportion Difference; NRC: Non-randomized controlled; RCT: Randomized controlled trials*.

Overall, interventions that were more effective in improving knowledge of CVD and risk factors mainly involved intervention components including health education through regular lecture, interactive workshop, group meetings, trainings by community volunteers and/or local healthcare staff [33,35,36,38,41,42,[44], [45], [46],^48^], health management advice and community support [36,38,45], and community wide interventions [37], [38], [39]. The three studies that showed non-significant difference in the effectiveness, tested interventions involving mainly promotion campaigns using posters, street theater, etc., counseling calls or take-home educational materials, distributed either electronically or printed. A three-arm trial by Fottrell et al. [48] found that participatory learning and action intervention were more effective than mHealth intervention in improving all components of knowledge related to causes, symptoms, and complications of diabetes. Furthermore, Silver et al. [39] examined the effectiveness using multiple arm of print materials, low-level TV and high-level TV campaign with no intervention, and found that both low- and high-level TV significantly improved the ability to name more than two signs of stroke. However, no significant improvement was observed for participants in the print group compared to no intervention.

All individual-randomized and 8 out of 9 NRC studies found that interventions were more effective than controls in improving at least one knowledge-related outcome measure. However, only one of four cluster randomized studies demonstrated the effectiveness of CBIs in improving CVD knowledge. On the other hand, five out of seven in LMICs and seven out of nine studies conducted in HICs found an improvement in the CVD knowledge in the intervention group compared with controls.

Two studies described the use of a one-to-one approach to deliver the proposed intervention package and in both of them the difference in change of knowledge level was found to be non-significant. One study [40] compared one-to-one vs. group meetings using a 2 × 2 factorial design and no significant difference was found across all comparison groups. Four out of five studies with interventions targeting high-risk groups and eight of 11 that targeted the general population showed a significantly higher increase in CVD related knowledge in the intervention group than controls. Two interventions involved primary care settings as part of the intervention center and both of them were effective in improving CVD knowledge.

Regarding the outcome measures, although two out of three studies showed effectiveness in improving overall knowledge related to diet and recommended level of fruit and vegetable intake, two studies that measured knowledge of portion size found no significant improvement post-intervention. Glasson et al. [38] also found that the community-based ‘Eat It To Beat It’ program increased the knowledge on recommended levels of fruit and vegetable intake but the effect on serving/portion sizes was not statistically significant.

## 4 Discussion

We performed a comprehensive systematic review of available literature on community-based preventive interventions and their effectiveness in improving CVD knowledge. Our findings support the potential of community-based preventive interventions to improve awareness and knowledge related to CVDs, risk factors and preventive mechanisms. As knowledge is an important prerequisite for behavioral change, CBIs could facilitate the primary prevention of CVDs and contribute to halt the continuing high burden of CVDs in various contexts. We found that the majority of studies, both NRCs and RCTs, demonstrated an improvement in CVD knowledge. Comparatively, interventions involving group lectures, training and/or workshops showed a larger effect than interventions through take-home self-learning materials, media campaigns and telephone calls. Interventions through mass media campaigns and/or posters were less effective in improving CVD knowledge in comparison with those targeted specific intervention populations. Furthermore, interventions that employed a one-to-one approach through telephone counseling or print materials showed no significant intervention effect. Interventions that targeted diet related knowledge showed improvement in the recommended level of fruit and vegetable intake but not knowledge about the portion size. Despite the fact that the CVD burden is higher in LMICs, studies quantifying the effect of interventions on knowledge levels are limited in low-income countries, particularly, no such studies were found in sub-Saharan Africa, where NCDs are the second most common cause of death [49].

Overall, our review indicated that CBIs are effective in improving CVD knowledge measured at one to three years of FU. Although limited to specific regions and contexts, previous reviews also indicated that CBIs are effective in improving CVD related knowledge, behavioral and metabolic risks [26,50]. One's health behavior depends on various personal, social and cultural factors apart from knowledge and perception, including capacity, self-efficacy, resources, and choice [51]. Hence, CVD preventive interventions should include strategies to translate the observed increase in knowledge to actual behavior change. Multicomponent interventions involving multi-disciplinary teams could help to translate an increase in knowledge to favorable intention and change in behavioral risks. With high levels of commitment and coordination, population-wide interventions might be feasible approaches in various contexts including resource-limited settings [52,53].

In this systematic review, the observed heterogeneity in the components of the intervention across various studies makes it hardly possible to depict certain aspects of the intervention attributable to the beneficial effect. Most intervention packages, however, used multi-faceted implementation approaches. Relatively, interventions that employed health education through either group lectures, workshops and/or training were more effective in improving CVD knowledge than interventions via media campaigns, telephone calls, and take-home materials. Likewise, a review by Van de Vijver et al. found that most successful interventions to improve behavioral and metabolic risk factors of CVD contain health education along with intensive training and coaching [26]. Several other studies also found educational interventions are effective in improving the level of CVD related knowledge and physical activity behavior [17,54,55]. Thus, besides other behavioral change activities, taking health education as one component of intervention could help to improve participant's CVD and risk factors knowledge which in turn facilitate adopting a healthy lifestyle.

Interventions that used a one-to-one approach showed less significant intervention effect in contrast to group-based interventions. A study by Imazu et al. also found that group-based intervention leads to a higher increase in knowledge than those individual-based [56]. Several other studies also demonstrated that group-based interventions are a more effective health education approach than one-to-one sessions [57,58]. Trief et al. investigated the effectiveness of group vs individual approach and found that group is more effective than solo contact in achieving behavioral change related to activity and diet [59]. This could be due to the presumption that in a group one can find the support and encouragement needed to acquire relevant knowledge [60]. Group-based interventions are more likely resource-saving in terms of total health professional or coaching staff hours per participant. We suggest future studies to evaluate the cost-effectiveness of group vs one-to-one approaches using comparable groups in different contexts.

On the other hand, interventions through take-home print or electronic materials were less effective compared to face-to-face training or lectures. Another previous study also showed that face-to-face education leads to better health outcomes than educational movies [61].

Despite the burden being higher in LMICs, our review found that such studies are scant in low-income countries particularly SSA, indicating unmet global health need and research effort, in which resources to conduct research are centralized in HICs. The larger share of the social and economic burden due to CVDs is in LMICs particularly sub-Saharan Africa, however, studies evaluating the effectiveness of CBIs are limited, challenging the development and implementation of evidence-based public health policies [62], [63], [64]. Therefore, more NCDs research centers need to be established in LMICs to investigate and evaluate sustainable preventive solutions through drawing upon existing research in HICs.

We also found that interventions were effective in increasing knowledge about fruit and vegetable intake whereas the change in knowledge about portion size was not statistically significant. Despite the knowledge of portion size being crucial to balance energy intake, most of the interventions were not effective. Therefore, innovative intervention strategies targeting practical knowledge of portion size are needed to halt the CVD burden related to dietary habits.

The implications of this review for future research and public health practice are that CBIs are effective and helpful in improving knowledge, which is an integral part of CVD prevention and control programs. Such interventions need to be scaled up and implemented in various contexts particularly in LMICs to create wider health impacts. Nevertheless, for such interventions to be effective, it is imperative to contextualize interventions and to identify the optimal strategy and approach that fit the target population and outcome. Furthermore, community-based lifestyle interventions could be a possible candidate as a strategy for CVD prevention and control in resource-limited settings. Nevertheless, it is essential to evaluate the cost effectiveness of such interventions in comparison with other primary prevention strategies in different contexts.

Methodologically, some of the included studies had high or some concerns of bias, particularly bias arose from insufficient description of deviation from the intended intervention, bias due to the timing of randomization and participant recruitment and bias from confounding. Thus, future studies that test the effectiveness of CBIs should make note of the recruitment process, details of the intervention activities and participants involvement during the intervention duration. Furthermore, studies should give more emphasis to control/adjust for possible confounding during the design or analysis phase.

This review has some limitations that need to be considered when interpreting the findings. First, due to the heterogeneity in the outcome measures, presentation of findings, and inconsistent intervention approach, extensive meta-analysis was not feasible. Thus, the data presented in this review are predominantly narrative. Nevertheless, this review highlights evidence on the approach and effectiveness of CBIs in improving knowledge about CVD risk factors and preventive mechanisms. Second, our review is restricted to the English language, which might lead to language bias. Last, due to inadequate description of the intervention for some of the studies, it was not possible to attribute certain intervention activities to the observed effectiveness. The use of template for intervention description and replication (TIDieR) checklist and guide is highly recommended to facilitate replication of the study. In spite of these limitations, this review highlighted the importance of preventive lifestyle interventions using community and primary healthcare settings in order to increase CVD knowledge and in turn improve healthy lifestyle. Thus, with the growing burden of NCDs including CVDs, scaling up effective CBIs should be considered as the main component besides pharmacologic intervention.

## 5 Conclusions

CBIs targeting to improve knowledge related to CVD risks and preventive mechanisms are promising to bring the intended change. The most effective interventions employed health education through workshops, training, group meetings, and counseling via primary healthcare or community volunteers. Such interventional studies are minimal in LMICs particularly no studies were available from sub-Saharan Africa. This indicates the need for further studies to contextualize and test the effectiveness of interventions in these resource-limited settings, where the CVD burden is disproportionately higher. In general, this review provides evidence to inform policy makers and public health practitioners to facilitate decision-making and prioritizing interventions for CVD prevention in various contexts. Thus, CBIs could play a key role in CVD prevention programs through improving CVD related knowledge besides other intervention strategies.

## Funding

The SPICES project in Belgium supports this study, which received funding from the European Commission through the Horizon 2020 research and innovation action grant agreement No 733356. The funder had no role in the design, decision to publish, or preparation of the manuscript.

## CRediT authorship contribution statement

**Hamid Yimam Hassen:** Conceptualization, Writing – review & editing, Investigation, Data curation, Writing – original draft, Validation. **Rawlance Ndejjo:** Investigation, Writing – review & editing, Validation. **Jean-Pierre Van Geertruyden:** Data curation, Supervision, Writing – review & editing, Validation. **Geofrey Musinguzi:** Supervision, Writing – review & editing, Validation. **Steven Abrams:** Conceptualization, Writing – review & editing, Data curation, Supervision, Validation. **Hilde Bastiaens:** Conceptualization, Writing – review & editing, Data curation, Supervision, Validation.

## Declaration of Competing Interest

The authors declare no competing interests.

## Appendix Supplementary materials

Image, application 1
