# Supplementary material for: Type and effectiveness of community-based interventions in improving knowledge related to cardiovascular diseases and risk factors: A systematic review
Source: Am J Prev Cardiol. 2022 Apr 6;10:100341. doi: 10.1016/j.ajpc.2022.100341 (PMC9035404; doi:10.1016/j.ajpc.2022.100341)
Supplement: Supplementary file 1 [file mmc1.pdf]

Supplementary Material: Type and effectiveness of community-based interventions in improving knowledge related to cardiovascular diseases and risk factors: A systematic review

Hamid Yimam Hassen[a*], Rawlance Ndejjo[a,d], Jean-Pierre Van Geertruyden[b], Geofrey Musinguzi[a,d], Steven Abrams[b,c¥], and Hilde Bastiaens[a,b¥]

[a] Department of Population Health and Family Medicine, Faculty of Medicine and Health Sciences, University of Antwerp, Antwerp, 2610, Belgium

[b] Global Health Institute, Faculty of Medicine and Health Sciences, University of Antwerp, Antwerp, 2610, Belgium

[c] Interuniversity Institute for Biostatistics and statistical Bioinformatics, Data Science Institute, Hasselt University, Diepenbeek, 3590, Belgium

[d] Department of Disease Control and Environmental Health, School of Public Health, Makerere University, Kampala, Uganda

*Correspondence to:

Hamid Yimam Hassen

Department of Population Health and Family Medicine, Faculty of Medicine and Health Sciences, University of Antwerp, Doornstraat 331 Wilrijk, 2610 Belgium

Hamid.hassen@uantwerpen.be

¥ - These authors share senior co-authorship.

Contents

Contents 2
Study characteristics 3
SWiM checklist 5
Cochrane risk of bias 7
Search strategy 10

Study characteristics

Table S1. Characteristics of included studies

Author, year, country<br>income per capita	Inter. duration (months)	Intervention setting	Participant age (range or mean (SD))	Sample size		Outcome measure	Summary of findings
				Int.	Cont.		
Individual randomized controlled studies							
Chao, 2012 (Chao, Wang et al. 2012), China [b]	18	PHC and home-based	60 or older	1163	1198	Health knowledge score	Compared with the control group, the management group demonstrated improvement on health knowledge score (P<0.01).
Lu, 2015 (Lu, Tang et al. 2015) China [b]	24	Community-based	40 to 75	240	120	Hypertension knowledge score	After the 2-year health education intervention, there were statistically significant increases in hypertension-related knowledge scores in all the three intervention groups. However, the increase was significantly greater in the interactive education workshop group 3 (mean score increased from 3.4 to 8.6) than in the regular lecture group 2 (mean score increased from 2.7 to 6.6) or self-learning reading group 1 (mean score increased from 3.6 to 5.8).
Brown 2002 (Brown, Garcia et al. 2002), USA [a]	12	Community-based	35 to 70	126	126	Diabetes knowledge scores	Experimental groups showed significantly higher diabetes knowledge scores at 12 month (Adjusted MD: 2.37, $p<0.001$, effect size = 0.07).
Cluster-randomized controlled studies							
Fottrell, 2019 (Fottrell, Ahmed et al. 2019), Bangladesh [b]	14	Community-based	30 or older	7610	3892	Diabetes knowledge	Both interventions were associated with improvements in diabetes knowledge (effect sizes are for individual items).
Joshi, 2012 (Joshi, Chow et al. 2012), India [b]	24	Combined (PHC, community)	30 or older	592	543	Knowledge 6 key health behaviors related to CVD	There was no detectable effect of the health promotion intervention on the knowledge about 6 lifestyle factors affecting CVD risk (p 0.15).
Resnicow, 2005 (Resnicow, Jackson et al. 2005), USA [a]	12	Community-based	18 to 86	639	267	Knowledge of portion size	There were no significant treatment × time effects for knowledge of portion size.
Simmons, 2015 (Simmons, Prevost et al. 2015), India [b]	12	Community-based	Mean ~ 65	group-330; individual-325; combined-322	322	Diabetes Knowledge	There was no significant intervention effect on diabetes knowledge (MD: 0.17; 95%CI: -0.21, 0.54)
Non-randomized controlled studies							
Huang, 2011 (Huang, Hu et al. 2011), China [b]	36	Community-based	35 and above	826	806	Knowledge and perceptions on HTN, dietary and lifestyle behaviors	Significantly, more participants in the intervention group responded correctly to the questions than those in the control group.

Kloek, 2006 (Kloek, van Lenthe et al. 2006), Netherlands [a]	24	Community-based	18 to 65	1426	1355	Fruit and vegetable knowledge score	A statistically significant effect was found in favor of the intervention neighborhoods with respect to the fruit and vegetable knowledge score.
Lv, 2014 (Lv, Liu et al. 2014) China [b]	24	Combined (PHC, Community)	18 to 64	1016	1000	Tobacco-, diet-, and PA-related knowledge	Over the 2-year intervention period, tobacco-related knowledge and beliefs improved in the intervention and comparison areas. The improvements were significantly larger in the comparison area (mean score from 5.21 to 6.38) than in the intervention areas (mean score from 4.97 to 5.74). The score for diet-related knowledge and belief declined from 1.98 to 1.68 in the intervention areas and did not change in the comparison area. The score for PA-related knowledge and belief declined slightly in the intervention (from 5.38 to 5.04) but not in the comparison area.
Nishtar, 2007 (Nishtar, Badar et al. 2007), Pakistan [b]	12	Community-based	18 to 65	288	304	Knowledge about CVDs and their prevention	The proportion who were active post-intervention was higher among intervention group than controls (p=0.007).
Silver, 2003 (Silver, Rubini et al. 2003), Canada [a]	24	Television advertisement	18+	392+412+403	410	Knowledge of >2 Warning Signs of Stroke	In communities exposed to television advertising, ability to name the warning signs of stroke increased significantly. There was no significant change in the community receiving print (newspaper) advertising, and the control community experienced a decrease.
Miyamatsu 2012 (Miyamatsu, Kimura et al. 2012), Japan [a]	12	TV campaign	40 to 70	968	971	Knowledge about early symptoms of stroke	After the 1-year television campaign, knowledge was significantly improved only in the campaign area (campaign area, 63%; 95% CI, 60%–66%; control area, 51%; 95% CI, 48%–54%).
Bertera 2014 (Bertera 2014), USA [a]	36	Community-based	55 or above	212	217	Knowledge related to diabetes and high blood pressure	Post-assessment scores on knowledge of blood pressure management techniques improved significantly compared with preassessments (p < .001, paired t test).
Glasson 2013 (Glasson, Chapman et al. 2013), Australia [a]	36	Community-based	18 or above	700	701	Knowledge of recommended intakes for fruit and vegetables, and knowledge of serving size	The program achieved improvements in knowledge of recommended intakes for fruit and vegetables and some positive changes in knowledge of serving size for vegetables.
Saito 2018 (Saito, Oguma et al. 2018), Japan [a]	24	Community-based	20 or above	391	994	Awareness and PA guideline knowledge	More adults in the intervention group were aware of the PA guidelines than in the control group at baseline (adjusted difference between groups = 0.82% (95% CI: 0.33, 1.31)).

AOR: Adjusted Odds Ratio; CG: Control Group; ES: Effect size; IG: Intervention Group; MD: mean difference; ND: No Data; PD: Proportion Difference; PHC: Primary healthcare; NRC: Non-randomized controlled; RCT: Randomized controlled trials

SWiM checklist

Table S2: SWiM checklist

Table 1 | Synthesis Without Meta-analysis (SWiM) items: SWiM is intended to complement and be used as an extension to PRISMA			
SWiM reporting item	Item description	**Page in manuscript where item is reported**	Other*
Methods			
1 Grouping studies	**1a) Provide a description of, and rationale for, the groups used in the synthesis (eg, groupings of populations, interventions, outcomes, study design)**	**6**	
	**1b) Detail and provide rationale for any changes made subsequent to the protocol in the groups used in the synthesis**	**6**	
2 Describe the standardized metric and transformation methods used	Describe the standardized metric for each outcome. Explain why the metric(s) was chosen and describe any methods used to transform the intervention effects, as reported in the study, to the standardized metric, citing any methodological guidance consulted	**6**	
3 Describe the synthesis methods	Describe and justify the methods used to synthesize the effects for each outcome when it was not possible to undertake a meta-analysis of effect estimates	**6**	
4 Criteria used to prioritize results for summary and synthesis	Where applicable, provide the criteria used, with supporting justification, to select the particular studies, or a particular study, for the main synthesis or to draw conclusions from the synthesis (eg, based on study design, risk of bias assessments, directness in relation to the review question)	**5, 6**	
**5 Investigation of heterogeneity in reported effects**	State the method(s) used to examine heterogeneity in reported effects when it was not possible to undertake a meta-analysis of effect estimates and its extensions to investigate heterogeneity	**6**	
6 Certainty of evidence	Describe the methods used to assess the certainty of the synthesis findings	**6**	
7 Data presentation methods	Describe the graphical and tabular methods used to present the effects (eg, tables, forest plots, harvest plots)	**6**	
	Specify key study characteristics (eg, study design, risk of bias) used to order the studies, in the text and any tables or graphs, clearly referencing the studies included	**6**	
Results			
8 Reporting results	For each comparison and outcome, provide a description of the synthesized findings and the certainty of the findings. Describe the result in language that is consistent with the question the synthesis addresses, and indicate which studies contribute to the synthesis	**7, 8, 9**	
Discussion			

9 Limitations of the synthesis	Report the limitations of the synthesis methods used and/or the groupings used in the synthesis and how these affect the conclusions that can be drawn in relation to the original review question	**13**	

PRISMA=Preferred Reporting Items for Systematic Reviews and Meta-Analyses.
*If the information is not provided in the systematic review, give details of where this information is available (eg, protocol, other published papers (provide citation details), or website (provide the URL)).

Cochrane risk of bias

Table S3 (a): Cochrane risk of bias of included Randomized Controlled studies

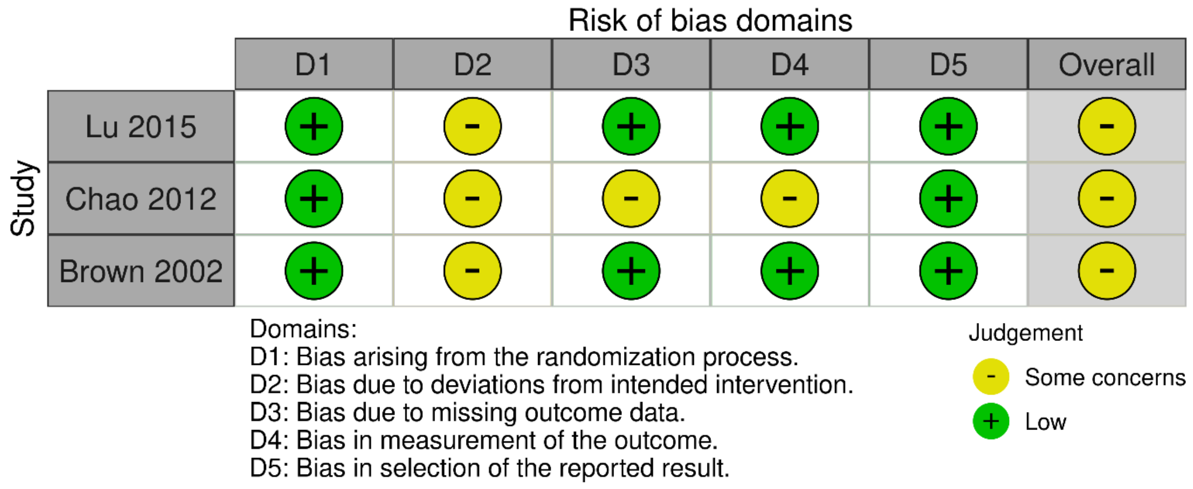

Fig S1 (a): Weighted summary of RoB for individual randomized controlled studies

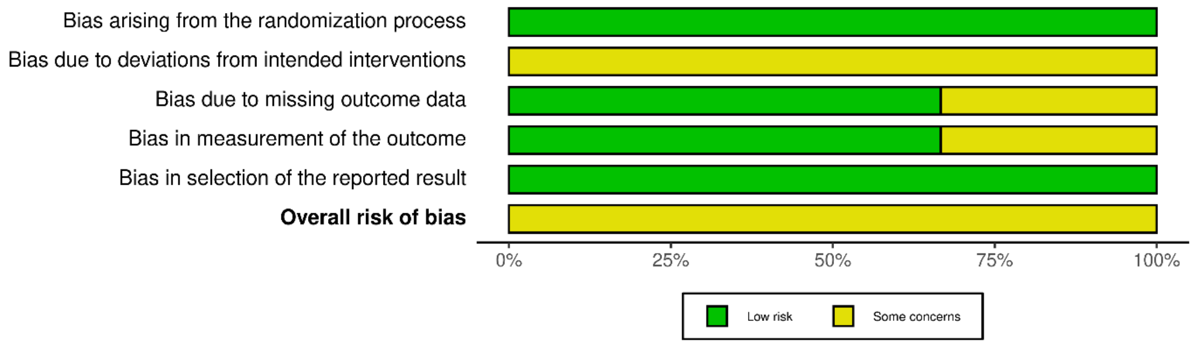

Table S3 (b): Cochrane risk of bias of included cluster randomized studies

Risk of bias domains

Study	D1	D1b	D2	D3	D4	D5	Overall
Fottrell 2019	+	-	-	+	-	+	-
Joshi 2012	+	-	X	+	-	+	X
Resnicow 2001	-	-	+	-	+	+	-
Simmons 2015	+	+	+	+	+	+	+

Domains:
D1 : Bias arising from the randomization process.
D1b: Bias arising from the timing of identification and recruitment of Individual participants in relation to timing of randomization.
D2 : Bias due to deviations from intended intervention.
D3 : Bias due to missing outcome data.
D4 : Bias in measurement of the outcome.
D5 : Bias in selection of the reported result.

Judgement
X High
- Some concerns
+ Low

Table S3 (c): Cochrane risk of bias of included non-randomized studies

Risk of bias domains

Study	D1	D2	D3	D4	D5	D6	D7	Overall
Lv 2014	-	+	+	-	+	+	+	-
Saito 2018	+	+	-	-	+	+	+	-
Glasson 2013	+	+	+	+	+	+	+	+
Bertera 2014	-	+	+	+	-	+	+	-
Silver 2003	-	+	+	+	+	+	+	-
Nishtar 2007	X	-	+	-	-	+	+	X
Huang 2011	X	-	+	-	+	+	+	X
Kloek 2006	+	+	+	+	+	+	+	+
Miyamatsu 2015	X	+	+	+	-	+	+	X

Domains:
D1: Bias due to confounding.
D2: Bias due to selection of participants.
D3: Bias in classification of interventions.
D4: Bias due to deviations from intended interventions.
D5: Bias due to missing data.
D6: Bias in measurement of outcomes.
D7: Bias in selection of the reported result.

Judgement
X Serious
- Moderate
+ Low

Fig S1 (a): Weighted summary of RoB for non-randomized controlled studies

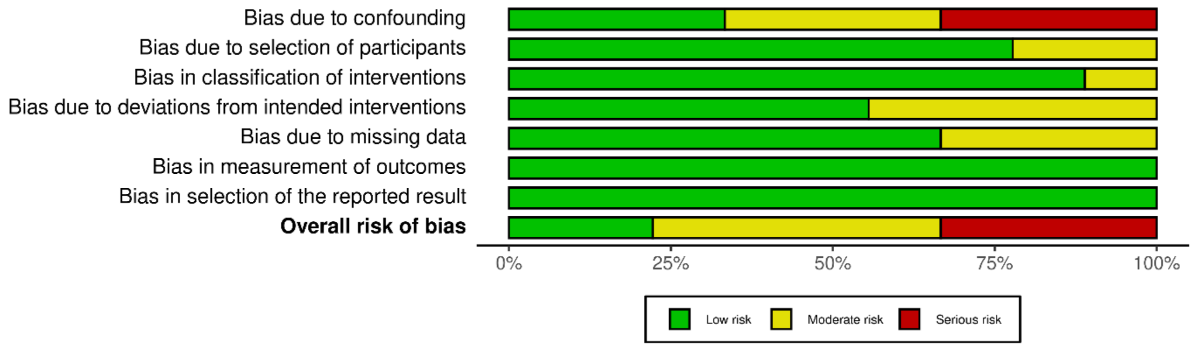

Search strategy

Box S1: Search strategy in Medline

#1	(“Community” OR “community-based intervention” OR “community-based” OR “community based” OR “community intervention” OR “population-based intervention” OR “population based” OR “population intervention” OR “community health” OR “community organisation” OR “community organization” OR “community program*” OR “Community level” OR “Community networks” OR “community health services” OR “home based” OR “community participation” OR “community-based research”)
#2	(Interven* OR strateg* OR approach* OR program* OR “health education” OR “health educ*” OR advise OR “raising awareness” OR counsel* OR “health promotion” OR “health campaign” OR “wellness program*” OR “mass media” OR “behaviour* change” OR “behavior* change” OR “lifestyle intervention” OR “lifestyle program*” OR “screening” “motivational interviewing” OR “risk scoring” OR refer* OR training OR “capacity building” OR “peer” OR “peer group” OR “community health worker” OR “CHW” OR “community health volunteer” OR “health worker*” OR “Community Health Extension Worker” OR “Health promoter” OR “Community Health Care Provider” OR “social support” OR “adherence support” OR “coaching” OR “self management” OR self-management OR “outreach” OR “home visit” OR “appointment reminders”)
#3	(“Cardiovascular disease” OR “CVD” OR “CVD risk” OR “cardiovascular disease prevention” OR “cardiovascular disease control” OR “stroke” OR “coronary heart disease” OR “heart diseas*” OR “heart failure” OR “kidney disease” OR “Cardiovascular risk factor” OR “hypertension” OR “raised blood pressure” OR diabetes OR “raised blood sugar” OR “cholest*” OR triglyceride OR HDL OR LDL OR “lipid profile” OR “metabolic syndrome” OR “body mass index” OR “BMI” OR “Overweight” OR “obesity” OR “obese” OR “waist circumference” OR “life style” OR “lifestyle” OR “alcohol” OR “tobacco” OR “smoking” OR “diet*” OR “nutrition” OR “food habit” OR “junk food” OR “fast food” OR “fruit” OR “vegetables” OR “five a day” OR “salt reduction” OR “physical inactivity” OR “physical activity” OR “exercise” OR “stress”)
#4	(“randomized controlled trial” OR “randomized” OR “randomised” OR “controlled study” OR trial OR RCT OR cluster OR CRT OR “comparative study” OR “quasi experimental study” OR “quasi-experiment” OR “experimental” OR “control group” OR “follow up” OR “prospective” or “retrospective” OR placebo OR random* OR “follow-up” OR “non-random*” OR “nonrandom*” OR “before after stud*” OR "before and after" or “time series” or “time-series” OR “interrupted time series” OR longitud* OR “controlled before” OR “pre-post” OR pretest OR posttest OR “pre intervention” or “post intervention”)
#4	#1 AND #2 AND #3 AND #4
#5	<u>Filters:</u> **year of publication:** (January 2000 to June 2019), **Language:** English **Age:** adults (18 and above) **population:** humans
